# Antenatal mindfulness intervention to reduce depression, anxiety and stress: a pilot randomised controlled trial of the *MindBabyBody* program in an Australian tertiary maternity hospital

**DOI:** 10.1186/s12884-014-0369-z

**Published:** 2014-10-25

**Authors:** Hannah Woolhouse, Kristine Mercuri, Fiona Judd, Stephanie J Brown

**Affiliations:** Healthy Mothers, Healthy Families, Murdoch Childrens Research Institute, WL5 Royal Children’s Hospital, Parkville, Melbourne, 3052 VIC Australia; Centre for Women’s Mental Health, Royal Women’s Hospital, Flemington Road, Parkville, Melbourne, 3052 VIC Australia; Department of Psychiatry, University of Melbourne, Level 1 North, Main Block, Royal Melbourne Hospital, Parkville, 3050 VIC Australia; General Practice and Primary Health Care Academic Centre, University of Melbourne, 200 Berkeley Street, Carlton, Melbourne, 3053 VIC Australia

**Keywords:** Mindfulness, Antenatal depression, Antenatal anxiety, Antenatal care, Stress in pregnancy

## Abstract

**Background:**

Mindfulness interventions to reduce psychological distress are well-suited to pregnancy, due to their brief and non-pharmacological nature, but there is a need for more robust evidence determining their usefulness. This pilot study was designed to explore the feasibility of a randomised controlled trial of a mindfulness intervention to reduce antenatal depression, anxiety and stress.

**Methods:**

The study was designed in two parts 1) a non-randomised trial targeting women at risk of mental health problems (a *selected* population) and 2) a randomised controlled trial (RCT) of a *universal* population. Process evaluation focused on feasibility of recruitment pathways, participant retention, acceptability of study measures, and engagement with mindfulness practices. Measurement of psychological distress was taken pre and post intervention through the Centre for Epidemiologic Studies Depression Scale Revised, the Depression Anxiety and Stress Scale-21, the State-Trait Anxiety Inventory, and the Perceived Stress Scale.

**Results:**

20 women were recruited to the non-randomised trial, and 32 to the RCT. Recruitment through a mailed study brochure at the time of booking-in to the hospital resulted in the largest number of participants in the RCT (16/32; 50%), and resulted in considerably earlier recruitment (50% in first trimester, 50% second trimester) compared to recruitment through the antenatal clinic waiting room (86% in second trimester, 14% third trimester). Over a third of women in the universal population scored above clinical cut-offs for depression and anxiety, indicating a sample with more symptomology than the general population. The most common reason for loss to follow-up was delivery of baby prior to follow-up (n = 9). In the non-randomised study, significant within group improvements to depression and anxiety were observed. In the intervention arm of the RCT there were significant within group improvements to anxiety and mindfulness. No between group differences for the intervention and ‘care as usual’ control group were observed.

**Conclusions:**

This small pilot study provides evidence on the feasibility of an antenatal mindfulness intervention to reduce psychological distress. Major challenges include: finding ways to facilitate recruitment in early pregnancy and engaging younger women and other vulnerable populations.

**Trial registration:**

Australian New Zealand Clinical Trials Registry ACTRN12613000742774 (31/10/2012).

## Background

Reducing perinatal distress is a vital public health goal [1]. Maternal anxiety, depression and stress (during and after pregnancy) have short and long term consequences for women’s health, affect mother-child interactions, and increase the risk of a range of ongoing emotional, behavioural and cognitive problems in children [2-4]. Importantly, it is not only diagnosable mental illness in mothers which is associated with poorer outcomes for children, but also a range of objective stressors and subjective stresses (including symptoms at sub-clinical levels) [5]. In this context, the optimal target population for intervention research aimed at reducing maternal psychological distress is unclear. Lumley and colleagues [6] described three potential intervention populations: *indicated, selected, and universal. Indicated* populations include women currently experiencing mental health symptoms, *selected* populations include women who are at an increased risk of experiencing mental health issues, and *universal* populations include all women in a particular group (i.e. all pregnant women). Limited health funding, and concerns about the relative benefits and potential risks of treatments (particularly pharmacological treatments) lead to most studies focusing on women with current mental health problems. However, given mounting evidence that a variety of common issues in pregnancy - such as relationship difficulties [7], intimate partner abuse [8], and even daily hassles [9] - are associated with adverse outcomes for children, it can be argued that interventions involving both selected and universal populations are warranted. Importantly, the evaluation of interventions targeting selected and universal populations has “substantial potential benefits for population health” [10].

Interventions comprising mindfulness practice are a relatively new approach to the prevention and treatment of mental health problems. Mindfulness is a quality of human consciousness that can be independently assessed, and is popularly defined by Jon Kabat-Zinn as “the awareness that arises from paying attention, on purpose, in the present moment and non-judgementally” [11]. Five factors have been identified as potential mechanisms of change through which mindfulness may impact on mental health outcomes: exposure, cognitive change, self-management, relaxation and acceptance [12]. Through mindfulness meditation, participants are encouraged to turn *towards* negative experiences which may have previously been avoided, thus providing an experience of exposure and an improved ability to tolerate negative states. Mindfulness practice may also lead to cognitive change, or more specifically, changes in the way one relates to thoughts [13]. By bringing awareness to our cognitive experiences, understanding can develop of the transient and sometimes inaccurate nature of our thoughts [14]. Consequently thoughts can be stripped of some their emotional and behavioural impact. Mindfulness training may also contribute to improved self-management. Increased awareness of physical and psychological experiences may allow individuals to utilise diverse coping strategies [15]. While relaxation is not an aim of mindfulness practice, it is a common outcome, and may play a part in the improvement of stress-related symptoms and physical disorders [16,17]. Acceptance (or non-judgement) is a central concept in the practice of mindfulness, and individuals are encouraged to accept all aspects of their experiencing, including thoughts, emotions, and physical sensations. The act of acceptance is considered valuable in that it removes the need for maladaptive avoidance strategies which can occur when experiences are considered unacceptable.

Mindfulness training has formed the basis of numerous intervention programs which principally use meditation and movement to cultivate mindfulness in specific contexts. In particular, it has been used in the areas of stress reduction (*Mindfulness-Based Stress Reduction*) [15], depression and relapse prevention (*Mindfulness-Based Cognitive Therapy*) [18], and more recently in childbirth and parenting (*Mindfulness-Based Childbirth and Parenting*) [19]. The current intervention (*MindBabyBody*) was informed by these three key programs and was designed to be delivered within the constraints of a public maternity hospital setting. To this end, it had to be acceptable and accessible to women, and economically efficient within available hospital resources.

Evidence of the efficacy of mindfulness interventions in treating a range of mental health issues is growing [12,20], and they appear well-suited to pregnancy given their brief and non-pharmaceutical nature. Two small trials have examined the efficacy of mindfulness-based group programs during pregnancy. Vieten and colleagues [21] found that participation in an 8-week mindfulness intervention in the latter half of pregnancy resulted in significantly reduced antenatal anxiety compared to a wait-list control group. Large effect sizes were noted for these reductions. A more recent study in Australia [22] found that 75% of participants in a mindfulness treatment group experienced a decrease in stress symptoms, and 67% showed positive change in levels of stress and self-compassion at three-month follow-up. All mindfulness studies to date in the antenatal period have called for further randomised controlled trials with larger samples and longer follow-up [19,21-23].

Prior to any large-scale evaluation study, a thorough process of piloting and feasibility work is advised by the Medical Research Council Guidance on Developing & Evaluating Complex Interventions [24]. Such developmental work prior to large-scale randomised controlled trials ensures that interventions are feasible in current systems, acceptable to participants, and can be delivered as intended [25].

The current study was designed to inform the development of a randomised controlled trial (RCT) evaluating a mindfulness-based group antenatal intervention to reduce perinatal stress, anxiety, and depression. The pilot aimed to explore feasibility issues including the appropriate patient population to target, recruitment, retention, engagement with mindfulness practices, and acceptability and discriminant ability of study measures. In particular we aimed to test the feasibility and acceptability of three universal recruitment strategies, and compare these to a targeted recruitment approach.

## Methods

### Design and population

The study was conducted at the Royal Women’s Hospital (the Women’s), a tertiary level maternity hospital in Melbourne, Australia. In 2011, the Women’s introduced mindfulness based group sessions (the *MindBabyBody* program) as a treatment option for women who were currently experiencing, or who were deemed to be at risk of stress, anxiety and depression. Women were referred for treatment in this program by midwives or obstetricians providing care in the antenatal setting.

The current pilot study expanded this clinical work and was designed in two parts:

1. A *non-randomised trial of a selected population -* open to women identified as at risk of perinatal stress, depression, or anxiety.
2. A *randomised controlled trial of a universal population* - open to all English-speaking women booked to give birth at the Royal Women’s Hospital.

The following inclusion criteria applied to all women in the study: booked in to give birth at the Women’s; >10 weeks gestation; 18 – 50 years old. Exclusion criteria were: >34 weeks gestation, current substance abuse; severe suicidal ideation, and insufficient fluency in English to participate in mindfulness sessions and complete written questionnaires. Women identified by their midwife (or another health professional) as being at risk of mental health problems were all allocated to the non-randomised trial (i.e. the selected population).

### Recruitment

#### Recruitment of the selected population to the non-randomised trial

Recruitment to the non-randomised component of the study involved referral by antenatal care practitioners in the antenatal clinic following recognition that the woman was ‘at risk’ of perinatal anxiety or depression. This was based on identification of past history or family history of mental health problems, or current psychosocial stressors and/or poor social supports. Following a referral, the group facilitator (KM) initiated contact with the potential participant and discussed the intervention in more detail. Women referred to the program were invited to take part in the research project, but were able to participate in the *MindBabyBody* program if they declined to be involved in the data collection which was part of the research. The program facilitator then provided the potential participant with a Study Recruitment Pack, including the baseline questionnaire, consent form, and a reply paid envelope for returning documents directly to the research team.

#### Recruitment of the universal population to the randomised trial

Recruitment to the RCT occurred via three pathways.

1. *Research assistant attending antenatal booking clinic*
  A female research assistant (HW) approached women attending the Royal Women’s Hospital antenatal clinic, and invited them to take part in the *MindBabyBody* pilot study, described as an evaluation of a group program designed to ‘help you reduce stress and manage your mood’ during pregnancy and the postnatal period. Women who expressed interest were provided with a Study Recruitment Pack, and invited to complete study materials and return them directly to the research team.
2. *Recruitment via a study brochure mailed to women at time of booking in*
  A study information brochure was included with information sent to women when they made initial contact with the Women’s to book in to give birth at the hospital. The brochure provided information about the study and invited women to contact the study investigator directly if they were interested in participating. A Study Recruitment Pack was sent to all women who inquired about the study, with study materials then mailed directly back to the research team.
3. *Recruitment via childbirth education and physiotherapy classes*
  Staff members responsible for childbirth education classes, and antenatal physiotherapy classes were provided with information about the program, and encouraged to pass on study brochures to women attending their classes. Through the study information brochure, women were invited to contact the study investigator directly if they were interested in participating, and were then sent a Study Recruitment Pack in the mail, which was returned directly to the research team via the reply paid envelope.

### Randomisation in the RCT

Following return of study materials, participants in the RCT were randomised either to participate in the 6-week *MindBabyBody* program, or to ‘Care as Usual’. Participants were randomised through the use of sealed sequential envelopes provided by the Clinical Epidemiology and Biostatistics Unit at Murdoch Childrens Research Institute. Participants were contacted via telephone to inform them of the randomisation result, and when necessary, to make arrangements for participation in the *MindBabyBody* intervention.

### Outcome measures and data collection

The study included assessment of clinical outcomes through self-report questionnaires (stress, depression and anxiety), process outcomes, and a small number of in-depth face-to-face interviews to assess participant experiences in the program. Demographic characteristics (age, number of previous children, marital status, employment status, highest level of education, and country of birth) were collected in the baseline questionnaire.

The Depression, Anxiety and Stress Scale-21 (DASS) [26] was included in questionnaires, providing separate scores for depression, anxiety and stress. This measure has demonstrated good psychometric properties in both clinical and non-clinical samples [27-29]. Categorical scoring options are provided, with scores ≥14 indicating moderate depression, scores ≥10 indicating moderate anxiety, and scores of ≥19 indicating moderate stress.

Depression was additionally measured by the Centre for Epidemiologic Studies Depression Scale Revised (CES-D) [30] a 20-item scale of depressive symptoms which has been used previously in antenatal populations [21,31-33]. This scale has demonstrated excellent psychometric properties, including high internal consistency, strong factor loadings, and theoretically consistent convergent and divergent validity [34]. A score of ≥16 is used to indicate clinical levels of depression.

Anxiety was additionally measured by the State subscale of the 40-item State-Trait Anxiety Scale (STAI) [35]. This scale includes 20-items measuring an individuals’ current level of self-reported anxiety. This anxiety measure has been widely used with pregnant populations [21,36,37], and has demonstrated good psychometric qualities [38]. A score of ≥40 is used to demonstrate clinical levels of state anxiety [35].

Stress was additionally assessed by the Perceived Stress Scale (PSS) [39]. This 10-item scale measures the extent to which the participant views the situations in their life as unpredictable or out of their control. Good psychometric qualities have been previously demonstrated [40,41] and the measure has been used in pregnant samples [21,42,43]. This scale is used as a continuous variable, with no set clinical cut-off, but higher scores indicate higher levels of stress.

Mindfulness was measured by the Five-Factor Mindfulness Questionnaire (FFMQ) [44]. This is a 39-item questionnaire which assess five factors of mindfulness: nonreactivity, observing, acting with awareness, describing and nonjudging. The FFMQ has shown good construct validity, and reliability [45,46].

#### Process evaluation

Process evaluation examined feasibility issues including: number of participants recruited via alternative pathways; participant characteristics according to recruitment pathway; study retention from enrolment to follow-up questionnaire (including reasons for withdrawal); engagement with mindfulness practices; and acceptability of study measures. To ascertain the recruitment pathways women followed to join the study, they were asked two questions in the baseline questionnaire: 1) Where did you first hear about the *MindBabyBody* pilot study? and 2) When did you make the decision to take part in the *MindBabyBody* pilot study? The recruitment pathway was recorded as the participant’s response to the second question.

#### Face-to-face qualitative interviews with *MindBabyBody* participants

Study consent forms allowed for participants to provide additional consent for a face-to-face interview about their experiences in the *MindBabyBody* intervention. A convenience sample of four women who consented to this were invited to take part in a face-to-face interview at the Women’s. The interview was presented as an opportunity to reflect on their experiences and learnings in the program, and a chance to give program organisers feedback on practical aspects of the intervention such as the timing, length and content of sessions. The interviews were conducted by HW or SB. All interviews were digitally recorded and transcribed verbatim.

Ethics approval for the study was obtained from the Royal Women’s Hospital Human Research Ethics Committee (12/23), and the Royal Children’s Hospital Human Research Ethics Committee (32200A). The pilot trial was registered with the Australian and New Zealand Clinical Trials Registry (ACTRN12613000742774; 31/10/2012).

### Intervention

The *MindBabyBody* program is a 6-session mindfulness-based group therapy program developed specifically for pregnancy by Investigator KM. Participants are introduced to the mindfulness approach and strategies, including formal and informal mindfulness practices, mindful movement, and cognitive exercises. The sessions took place on weekdays, on-site at the Women’s. Two alternative timings were offered (during work hours, and in the evening). Participants did not receive any remuneration (such as travel or parking costs) for participation in the program. Sessions ran for two hours, and occurred weekly for six weeks. Participants were encouraged to attend all sessions, but were considered to have completed the program if they attended four of the six sessions. The group facilitator was a female mental health professional (psychiatrist/psychologist) with specific training in the facilitation of mindfulness groups. The facilitator was responsible for noting and responding to any emotional issues which arose for group participants during the course of the program, and for providing appropriate referral pathways where necessary.

Key features of the 6-session program are described in the program manual which was developed as part of the pilot study [47]. This outlines the main activities for each session, the time allocated for each activity, and the purpose of specific activities. Briefly, each session includes a formal meditation practice (15–20 mins), a discussion of home mindfulness practices, the mindful movement sequence, a weekly discussion topic, and a breathing space. Each week suggestions were given for home practise with repetition emphasised as a significant reinforcer of new skills. Week 1 included time to get to know each other, an introduction to mindfulness and a mindful breathing practice. Week 2 focused on mindfulness of the body, including a body scan, and the importance of the body in communicating with babies. Week 3 introduced ideas related to mindfulness of pain (physical and emotional), and how this might be relevant to labour. Week 4 focused on an ice meditation [19] where participants were given experience practicing mindfulness of painful sensations. Week 5 focused on mindfulness of thoughts, and Week 6 was centred on self-compassion, and the use of mindfulness skills in motherhood. Initially programs were offered during business hours (i.e. 9 am-11 am on a weekday), but part-way through recruitment, it became apparent that this option was not possible for a sizable proportion of women. We subsequently made the decision to offer some programs after hours (i.e. 6 pm-8 pm on a weeknight).

The control group was assigned to receive ‘care as usual’ and therefore did not take part in the *MindBabyBody* program. ‘Care as usual’ involves regular appointments with midwives in the antenatal clinic. These appointments include routine psycho-social screening, and the monitoring of mental and physical health by primary care professionals, with referral to specialised health professionals where appropriate.

### Data collection

Self-report questionnaires were collected at enrolment and at: a) completion of the 6-week program for participants who took part in the *MindBabyBody* group, and b) 8 weeks post enrolment for the ‘care as usual’ participants.

#### Data analysis

Quantitative data were analysed using STATA version 13. Descriptives are presented for socio-demographic characteristics. Baseline mental health data (mean, standard deviation) are presented for outcomes for all women who enrolled in the study, and the selected and universal population were compared using a two-sample t-test. Pre-post analysis for the non-randomised trial was conducted for those participants who had returned baseline and follow-up questionnaires, via paired sample t-tests. Analysis of outcome data for the randomised controlled trial involved paired-sample t-tests to explore within group differences over time, and comparison of the post-program mean scores for the intervention group and the care as usual group, via two-sample t-tests. However it should be noted that the current pilot study is not adequately powered to detect differences in these outcomes.

Qualitative interview transcripts were analysed using Interpretative Phenomenological Analysis (IPA) [48]. The aim of IPA is to conduct an in-depth exploration of how individuals make sense of their personal and social world. At the heart of the IPA approach is an emphasis on the ‘lived experience’ of study participants, and an attempt to understand their perception of the world around them. It is therefore a valuable method for bringing women’s own voices to light [49]. Data was analysed following the step-by-step procedures recommended by Smith [48]. The initial stages of data analysis involved looking at each interview transcript individually, making comments in the left hand margin on emerging areas of interest including: connections, preliminary interpretations, the use of language, a sense of the person being interviewed, and contradictions. During a second read-through of the transcript, possible emergent themes were noted in the right-hand margin. As additional transcripts were analysed, emergent themes were noted down, and connections between them were sought. A detailed table of themes was created, and ordered coherently, with related themes linked together and given a suitable title, and sub-ordinate themes listed below. The identified themes were then translated into a coherent narrative.

### Details of ethics approval

The current *MindBabyBody* pilot study was approved by the relevant human research ethics committees in both the Royal Women’s Hospital (Project 12/23) and the Royal Children’s Hospital (Project 32200 A).

## Results

### Flowchart of participation

**Figure 1:**
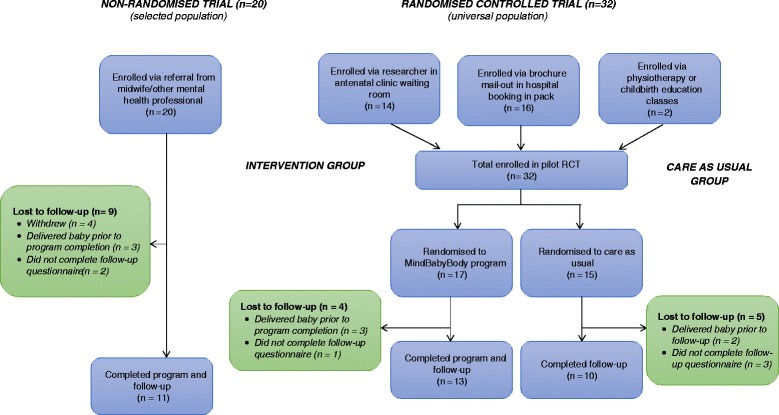
**Flowchart of participation.**

The flowchart of participation is presented in Figure 1. Over an 8-month period, 20 participants were recruited to the non-randomised trial. Over the same time period, 32 participants were recruited to the randomised controlled trial. HW attended the antenatal clinic waiting room on a total of six occasions, for four hours on each occasion, distributing approximately 50 Study Information Packs, a recruitment strategy which yielded 14 participants. A total of 2500 brochures were mailed out in booking in packs over the 8-month period, yielding 16 participants. Recruitment via physiotherapy or childbirth education classes over this time period resulted in two participants.

The rate of loss to follow-up in the non-randomised trial was 45% (9/20), and in the RCT was 28% (9/32). 33% (5/15) of participants in the ‘care as usual’ group were lost to follow-up, compared to 24% (4/17) in the intervention group. The most common cause of loss to follow-up was delivery of baby prior to completion of the intervention or prior to return of study measure (n = 8). In the non-randomised trial, four women withdrew from participation in the intervention part-way through the *MindBabyBody* program, and did not complete follow-up questionnaires. Six women in the overall study who had completed the *MindBabyBody* program did not return follow-up measures (two in the non-randomised trial and four in the RCT).

### Socio-demographic characteristics

Participants in the pilot ranged in age from 19 to 45 years (Mean = 32.89, SD = 0.63). Table 1 reports the demographic characteristics of study participants, for: 1) the non-randomised study of selected participants and the randomised trial of unselected participants, and 2) the intervention and ‘care as usual’ groups in the RCT. Compared to women in the non-randomised study, there was a higher proportion of women in the RCT who were having their first baby (*t* (50) = −2.42, *p* = 0.02), and who enrolled in the research study earlier in pregnancy (*t* (50) = −4.13, *p* <0.01). Some differences were apparent between the intervention and ‘care as usual’ groups, with women randomised to the intervention appearing younger, and more commonly having their first baby. Demographic characteristics according to recruitment pathway to the RCT (mailed brochure versus antenatal clinic; data not shown in table) indicated no differences except for the timing of recruitment. Recruitment through the mailed brochure resulted in considerably earlier recruitment (50% in first trimester, 50% in second trimester) compared to recruitment through the antenatal clinic waiting room (86% in second trimester, 14% in third trimester).

**Table 1:** **Baseline demographic characteristics**

	**Non-randomised trial (selected population) (n = 20)**	**Randomised trial (universal population) (n = 32)**	**Randomised trial - intervention group (n = 17)**	**Randomised trial – care as usual group (n = 15)**
**Age**				
Mean (SD)	33.70 (1.29)	32.39 (0.65)	30.81 (0.75)	34.08 (0.90)
**Parity**				
No prior births	10/20 (50.0)	27/32 (84.4)	16/17 (94.1)	11/15 (73.3)
1 or more prior births	10/20 (50.0)	5/32 (15.6)	1/17 (5.9)	4/15 (26.7)
**Employment status**				
Paid work	13/20 (65.0)	29/32 (90.6)	16/17 (94.1)	13/15 (86.7)
Not in paid work	7/20 (35.0)	3/32 (9.4)	1/17 (5.9)	2/15 (13.3)
**Education level**				
Higher university degree	5/20 (25.0)	14/32 (43.8)	6/17 (35.3)	8/15 (53.3)
University degree	10/20 (50.0)	13/32 (40.6)	7/17 (41.2)	6/15 (40.0)
Below university education	5/20 (25.0)	5/32 (15.6)	4/17 (23.5)	1/15 (6.7)
**Relationship status**				
Married	16/20 (80.0)	21/32 (65.6)	12/17 (70.6)	9/15 (60.0)
Living with partner	2/20 (10.0)	10/32 (31.3)	4/17 (23.5)	6/15 (40.0)
Single	2/20 (10.0)	1/32 (3.1)	1/17 (5.9)	0/15
**Born in Australia**				
Yes	8/20 (40.0)	16/32 (50.0)	7/17 (41.2)	9/15 (60.0)
No	12/20 (60.0)	16/32 (50.0)	10/17 (58.8)	6/15 (40.0)
**English first language**				
Yes	17/20 (85.0)	26/32 (81.3)	12/17 (70.6)	14/15 (93.3)
No	3/20 (15.0)	6/32 (18.7)	5/17 (29.4)	1/15 (6.7)
**Trimester at enrolment**				
First	0	8/32 (25.0)	3/17 (17.7)	5/15 (33.3)
Second	9/20 (45.0)	20/32 (62.5)	12/17 (70.6)	8/15 (53.3)
Third	11/20 (55.0)	4/32 (12.5)	2/17 (11.8)	2/15 (13.4)

### Baseline mental health

Table 2 shows a comparison of baseline mental health data for the selected and universal populations. The selected population had significantly higher depression, anxiety, and stress scores than the universal population. The universal population had higher scores on all subscales of the Five Factor Mindfulness Questionnaire, though these differences did not reach statistical significance.

**Table 2:** **Baseline mental health (selected versus universal population)**

	Non-randomised trial (selected population) (n = 20)*	Randomised trial (universal population) (n = 32)*	***Comparison***		
	***M (SD)***	***M (SD)***	***Mean difference (95% CI)***	***t (df)***	***p***
**Depression**					
CES-D depression	23.18 (8.04)	13.74 (8.68)	−9.43 (−14.57, −4.30)	−3.70 (46)	**<0.001**
DASS depression	12.95 (8.01)	6.88 (8.30)	−6.07 (−10.84, −1.30)	−2.56 (49)	**0.01**
**Anxiety**					
STAI state anxiety	48.39 (12.59)	35.83 (12.05)	−12.56 (−19.91, −5.20)	−3.44 (46)	**<0.001**
DASS anxiety	10.00 (7.54)	7.25 (7.48)	−2.75 (−7.12, 1.62)	−1.27 (49)	0.21
**Stress**					
PSS stress	21.80 (4.99)	17.06 (6.43)	−4.74 (−8.13, 1.35)	−2.81 (50)	**<0.001**
DASS stress	19.16 (7.25)	13.88 (10.24)	−5.28 (−10.67, 0.10)	−1.97 (49)	0.05
**Mindfulness**					
Nonreactivity	18.74 (5.25)	20.00 (6.04)	1.23 (−2.16, 4.62)	0.73 (47)	0.47
Observing	21.68 (4.97)	24.03 (6.01)	2.35 (−0.98, 5.67)	1.42 (47)	0.16
Acting with awareness	23.68 (3.51)	26.27 (5.97)	2.58 (−0.46, 5.63)	1.71 (47)	0.10
Describing	26.37 (4.79)	27.29 (6.70)	0.92 (−2.63, 4.47)	0.52 (48)	0.60
Nonjudging	23.32 (5.96)	25.87 (6.39)	2.55 (−1.13, 6.23)	1.40 (47)	0.17
*Full scale*	113.79 (14.76)	123.07 (20.74)	9.28 (−1.79, 20.35)	1.69 (46)	0.10

*Denominators may vary due to missing numbers.

NB. Significant differences are presented in bold.

Table 3 shows the proportion of participants in each group who scored above clinical-cut-offs for the scales with these available, and the range of scores on each scale. A considerably higher proportion of participants in the selected population scored above clinical cut-offs for depression, anxiety and stress. However, around a third of participants in the universal sample also fell into the clinical range. For each of the scales with clinical cut-offs (CESD, STAI State anxiety, DASS scales) the selected population baseline mean was above the clinical cut-off, indicating significant symptoms of stress, anxiety and depression. In the universal population, none of the baseline mean scores were above the clinical cut-off point.

**Table 3:** **Clinical cut-offs: baseline mental health**

		***Non-randomised trial (selected population)***			***Randomised trial (universal population)***		
	***Clinical cut-off***	***Above clinical cut-off***			***Above clinical cut-off***		
		***n***	***%***	***Range***	***n***	***%***	***Range***
CESD depression	≥ 16	12/17	71	10-34	10/31	32	2-32
DASS depression	≥ 10	12/19	63	0-28	8/32	25	0-36
STAI state anxiety	≥ 40	13/18	72	26-68	9/30	30	20-66
DASS anxiety	≥8	11/19	58	2-28	10/32	31	0-28
DASS stress	≥15	14/19	74	2-32	12/32	38	0-34

### Pre-post comparison of outcome data

#### Non-randomised study outcome data

Table 4 shows the results of paired t-tests comparing pre and post outcome data from the non-randomised study of selected participants. Significant improvements were noted on the DASS-21 depression scale (Cohen’s d = 0.6), the CES-D (Cohen’s d = 0.7) and the STAI state scale (Cohen’s d = 0.8). Stress scores were reduced at post-program, but the difference was not statistically significant. Mindfulness scores increased significantly on two of the five FFMQ subscales: acting with awareness (Cohen’s d = 0.9) and describing (Cohen’s d = 0.8).

**Table 4:** Pre-post outcome data for the non-randomised trial (selected population; n = 11)*

	***Pre-program M (SD)***	***Post-program M (SD)***	***Mean difference (95% CI)***	**t (df)**	**p**
**Depression**					
CES-D depression	24.60 (8.19)	18.20 (9.13)	6.40 (0.54, 12.26)	2.47 (9)	**0.04**
DASS depression	13.80 (7.74)	9.60 (6.10)	4.20 (1.38, 7.01)	3.37 (9)	**0.01**
**Anxiety**					
STAI state anxiety	49.67 (15.22)	39.33 (8.26)	10.33 (0.47, 20.20)	2.42 (8)	**0.04**
DASS anxiety	10.20 (2.52)	7.20 (4.54)	3.00 (−1.88, 7.88)	1.39 (9)	0.20
**Stress**					
PSS stress	22.46 (5.79)	17.18 (5.84)	5.27 (−0.86, 11.41)	1.91 (10)	0.09
DASS stress	21.20 (9.00)	16.60 (7.24)	4.60 (−0.45, 9.64)	2.06 (9)	0.07
**Mindfulness**					
Nonreactivity	18.00 (5.83)	20.64 (5.71)	−2.64 (−6.48, 1.20)	−1.53 (10)	0.16
Observing	22.73 (3.17)	25.55 (4.57)	−2.82 (−5.65, 0.01)	−2.22 (10)	0.05
Acting with awareness	24.09 (2.91)	27.45 (4.30)	−3.36 (−5.43, −1.30)	−3.63 (10)	**0.01**
Describing	26.27 (4.56)	29.91 (6.24)	−3.64 (−6.64, −0.63)	−2.69 (10)	**0.02**
Nonjudging	25.45 (5.87)	27.18 (6.15)	−1.73 (−5.02, 1.57)	−1.17 (10)	0.27
*Full scale*	116.55 (13.27)	130.73 (19.83)	−14.18 (−23.13, −5.23)	−3.53 (10)	**0.01**

*Denominators may vary due to missing numbers.

NB. Significant differences are presented in bold.

#### RCT outcome data

Table 5 reports the within group comparison of pre and post outcome data for the RCT. Within group comparisons showed some evidence of intervention effect. For the intervention group, all post-program mental health scores improved, with changes on the DASS-21 anxiety subscale reaching statistical significance (Cohen’s d = 0.7). On the FFMQ, the intervention group showed significant increases on two of the five subscales of the FFMQ: observing (Cohen’s d = 1.1) and describing (Cohen’s d = 0.3). No significant changes on outcome measures over time were observed in the control group. A between group comparison of the post-program means for the intervention and care as usual group was conducted via two-sample t-tests, and no significant between group differences were found.

**Table 5:** **Within group comparison for the RCT (universal population)**

	MindBabyBody intervention group (n = 13)*					Care as usual group (n = 10)*				
	***Pre-p*** **r** ***ogram M (SD)***	***Post-program M (SD)***	***Within group comparison***			***Pre-p*** **r** ***ogram M (SD)***	***Post-program M (SD)***	***Within group comparison***		
			***Mean diff (95% CI)***	***t (df)***	***p***			***Mean diff (95% CI)***	***t (df)***	***p***
**Depression**										
CES-D depression	14.42 (10.05)	12.08 (4.17)	2.33 (−4.60, 9.27)	0.74 (11)	0.47	13.70 (8.00)	10.10 (8.72)	3.60 (−1.40, 8.60)	1.62 (9)	0.14
DASS depression	7.23 (6.66)	4.31 (3.64)	2.92 (−1.16, 7.01)	1.56 (12)	0.15	8.00 (11.20)	5.60 (8.32)	2.40 (−1.91, 6.71)	1.26 (9)	0.24
**Anxiety**										
STAI state anxiety	35.92 (14.11)	32.83 (7.08)	3.08 (−7.02, 13.19)	0.67 (11)	0.52	34.78 (11.51)	33.00 (12.78)	1.78 (6.55, 6.82)	0.81 (8)	0.44
DASS anxiety	8.62 (7.72)	4.62 (3.95)	4.00 (0.69, 7.31)	2.63 (12)	**0.02**	7.00 (8.34)	4.80 (5.90)	2.2 (−0.92, 5.32)	1.59 (9)	0.15
**Stress**										
PSS stress	17.92 (7.14)	16.54 (6.12)	1.38 (−4.16, 6.93)	0.54 (12)	0.60	16.90 (7.08)	14.40 (8.41)	2.50 (−1.38, 6.38)	1.46 (9)	0.18
DASS stress	16.15 (11.27)	12.92 (5.01)	3.23 (−3.74, 10.20)	1.01 (12)	0.33	13.40 (10.79)	9.00 (4.92)	4.40 (−2.89, 11.69)	1.37 (9)	0.20
**Mindfulness**										
Nonreactivity	20.58 (6.73)	21.83 (4.45)	−1.25 (−4.89, 2.39)	−0.76 (11)	0.47	19.70 (5.25)	19.80 (4.78)	−0.10 (−1.83, 1.63)	−0.13 (9)	0.90
Observing	24.67 (5.25)	29.92 (4.54)	−5.25 (−8.50, −2.00)	−3.55 (11)	**<0.001**	24.80 (7.08)	25.50 (7.28)	−0.70 (−4.82, 3.42)	−0.38 (9)	0.71
Act with awareness	25.75 (5.79)	27.17 (4.00)	−1.42 (−4.91, 2.08)	−0.90 (11)	0.39	25.90 (6.38)	27.00 (2.98)	−1.10 (−4.71, 2.51)	−0.69 (9)	0.51
Describing	26.38 (6.79)	28.62 (6.59)	−2.23 (−4.16, −0.30)	−2.52 (12)	**0.03**	31.20 (5.18)	31.40 (5.48)	−0.20 (−2.33, 1.93)	−0.21 (9)	0.84
Nonjudging	25.25 (7.06)	27.58 (5.20)	−2.33 (−7.16, 2.49)	−1.06 (11)	0.31	24.90 (3.41)	29.80 (5.12)	−4.9 (−10.11, 0.31)	−2.13 (9)	0.06
*Full scale*	121.55 (23.65)	134.55 (20.55)	−13.0 (−27.79, 1.79)	−1.96 (10)	0.07	126.50 (15.77)	133.50 (12.43)	−7.0 (−15.53, 1.53)	−1.86 (9)	0.10

*Denominators may vary due to missing numbers.

NB, Significant differences are presented in bold.

### Engagement with mindfulness practices

Participants were asked to practice formal mindfulness meditation on a daily basis. Most commonly, participants reported formal mindfulness practice on 3–4 days per week. There appears to be a slightly higher level of practice in the RCT compared to the non-randomised study. For example, in the RCT, 92.3% reported practice on 3 days/week or more, compared to 63.6% in the non-randomised study. Formal practice on 5–7 days per week was reported by 23.1% and 27.3% of the randomised and non-randomised participants respectively. Overall, 87% reported that they practiced for 6–10 minutes or longer during formal meditation practice. Informal mindfulness practice was reported on 4 days a week or more by over half the participants (55%).

The control group in the RCT was asked if they had practiced any formal meditation or prenatal yoga during their pregnancy, to assess for cross-contamination. 50% of control participants had been practicing prenatal yoga between the pre and post follow-up, but only 10% had practiced any formal meditation between the pre and post follow-up.

### Qualitative results

Face-to-face in-depth interviews were undertaken with four program participants (two who were recruited through their consulting midwife (participants C and A), one recruited via the mailed antenatal brochure (participant B), and one recruited in the antenatal clinic waiting room (participant D). The results of the interviews are presented here in four parts: *expectations and motivations; experiences in the group; engagement with mindfulness practices;* and *changes attributed to mindfulness practice*.

#### Expectations and motivations

Each of the women interviewed had no prior experience or knowledge of mindfulness meditation prior to participating in the *MindBabyBody* group, and entered the program unsure what to expect. The opportunity to ‘learn a new tool’ was a common motivation. Three of the four women had signed up to the program with the aim of managing specific mental health challenges.

I was trying to manage my symptoms sort of the best I could without medication, but the first three months were really, really hard. I didn’t know anything about mindfulness, basically I just wanted to get better….I was just happy to try anything that was put my way. (C).

One of the midwives suggested it, because of my history with panic attacks. (A).

Yeah I didn’t really have much of an idea what to expect or anything….just learning new ways of dealing with things, because in the past I have been a heavy drinker and that was how I coped when things got too much… And when I became pregnant that was no longer a release or an outlet for me. (B).

The fourth participant identified her main motivation as altruistic, with a strong interest in research and giving back.

I’m trained as a scientist so I saw this as a way to keep contributing to the science field…and I saw it as a way to prepare for the birth and motherhood beyond that. (D).

#### Experiences in the group

The experience of being in the group was identified as somewhat challenging initially, but ultimately enjoyable. Particularly in the first few sessions, when the group was forming, a sense of discomfort was identified.

I guess I was a little bit sort of uncomfortable, like I was very uncomfortable for everybody in the room I think, particularly the first session. (C).

I think with any group session the very first session was a little bit, not confronting for me but we hadn’t quite gelled as a group and that’s to be expected. (D).

Once this sense of group connection was developed, sharing time with other pregnant women was identified as enjoyable.

I had some reservations (after the first session). But from that point on, I really enjoyed it. (A).

#### Engagement with mindfulness practice

Individuals engaged with the mindfulness practice in different ways. Of particular note were the contrasting reactions to specific practices. The ice meditation, which encouraged participants to try out different mindful approaches to pain (generated by holding a handful of ice) was identified as very helpful by some, but unhelpful by others.

I didn’t like the pain meditation practice thing that we had. .. I realised that I would just get so overwhelmed by the physical sensation that my brain just wouldn’t behave. And that was a little bit frustrating and unsettling. (B).

We [participant and her partner] did the ice meditation together because he was kind of freaking out about what to do in labour…And so I thought it would be really good if we did the ice together….it was certainly helpful for us. Like I would recommend that I guess for couples that have trouble communicating. (C).

Another formal practice which was commented on frequently was the loving kindness meditation, which was identified as particularly challenging – and also helpful in revealing the difficulty of practicing self-compassion.

It definitely showed that you don’t put much love onto yourself (A).

I found the self-compassion one quite good because it’s, I think it was something I, we all sort of felt that we were guilty of not doing… that one probably stuck out quite a bit because I do tend to be quite, um, self-flagellating … but again it’s a bit difficult to put into practice. (B).

Despite these quite different responses to some of the practices, the interviewees didn’t feel that any specific exercises should be left out of the program. The introduction of a variety of exercises was considered a strength of the program because it allowed participants to pick and choose those which suited them best.

Engaging with the practices and setting up a regular mindfulness practice at home was also experienced in different ways. Some participants engaged more with formal practice, and some with the informal mindfulness of daily activities. Levels of engagement with home practice during participation in the group ranged from once or twice through the whole program, to every day.

My partner and I were both doing body scan at night before we went to bed, every night we did it. (C).

[Re formal practice] probably only once or twice during the whole program. So I really only practiced it in the group….Maybe once or twice the whole time… The mindfulness of daily tasks, that’s what I’ve been doing a lot of, like the mindful walking, and things like that. I do those every day. (A).

Setting up a regular, daily practice at home was identified as a major challenge by three of the four women identified.

I think the challenge was integrating it into the day, and it was the simple obstacles like my computer not working anymore and not having another way to play the meditations. (D).

I don’t know why but I always find a block with coming to the formal meditation, or setting aside time to do that sort of thing.... I have other things to do or I’d rather read this book or I’d rather be, my mind would rather be busy. (B).

#### Changes attributed to mindfulness practice

Women were asked directly to reflect on any changes they felt had occurred for them as a result of taking part in the *MindBabyBody* program. The four main themes identified were; reigning in unhelpful patterns; improvements to interpersonal relationships; and general improvements to quality of life; and improved sleep.

A strong theme running through the interviews and identified by women, was the way in which mindfulness practice had allowed them to ***reign in destructive patterns*** – both cognitive, emotional and behavioural. Rather than getting caught up in negative thoughts and emotions, and escalating them to damaging levels, women were able to step back, observe, and make more considered responses to challenging situations.

[The most helpful thing was] learning how to get a grasp of negative thought processes that make me angry - particularly with my partner - and not carrying through to crisis point I suppose…that was actually a really big one for me. Because I do tend to allow my thoughts to go down a path where I’m expecting the worst or I’m envisaging a very bad thing to happen in the end. …so it has helped me to reign those in and not stress so much about the things that might not even happen…That was very big. (B).

I found acknowledging how I feel about certain things in the moment and being really present allowed me to sort of deal with whatever emotions that in certain situations were brought up. But rather than letting that irritability just sort of build up and build up and then all of a sudden I feel depressed or I feel annoyed or something like that, you know, it’s sort of just okay. You know what I mean? When things, when I’m feeling something now I can go okay, and then I can just sort of contain it to what’s happening, which is good…. I think definitely having it as a tool to sort of stop things before they start is fabulous. (C).

This improved ability to reflect on difficult thoughts and emotions, and reign in unhelpful patterns had various flow-on benefits to ***interpersonal relationships***, including to intimate relationships, extended family relationships, and experiences at work.

A lot of it is to do with my relationship with my partner as well….I think it has improved our relationship. Just through me being able to get a hold of those things [unhelpful thought patterns]. (B).

If my boss is giving me criticism then I, or I feel like I’ve missed something or done something wrong, then I get quite an aggressive voice in my head beating me up about it. Um, and I think the, being able to observe that response and acknowledge and move on kind of thing, was, meant that the scale of a problem, or the scale of the feeling associated with something going wrong was much less. (D).

Generalised improvements to mood and ***quality of life*** were also noted.

I’ve got to say the mindfulness in general has pretty much changed my life. It has helped me to be far more in tune and more relaxed about being a mum and baby and that sort of stuff as well. (C).

It’s definitely impacted on my mood… [Regarding informal practice, walking and doing the dishes] Well I used to really dread doing those things, and now I don’t mind doing them at all. It really helps me wind down. (A).

Many of the participants reported using formal mindfulness practices (particularly the Body Scan) to help them get to sleep.

It definitely helped me get to sleep yeah. I think that really helped, maybe on a subconscious level or I’m not really sure but it really did help me. (C).

I did about two of them at home [Body Scans], and it really helped me to get to sleep which was good. (A).

During the interview, each woman was asked how (or if) they planned to continue using mindfulness practices now the program had finished. Each of the women interviewed intended to continue with both formal and informal mindfulness practice.

## Discussion

### Key findings

The current study aimed to test the feasibility of a 6-session mindfulness intervention conducted in pregnancy through the antenatal care services of a large tertiary level maternity hospital. In addition we aimed to assess women’s experiences of participation in the program, and the acceptability of outcome measures. The study was designed to inform the development of a randomised controlled trial evaluating a mindfulness-based group antenatal intervention to reduce perinatal stress, anxiety, and depression.

### Target population

The findings of our study do not provide a conclusive answer on the optimal population to target for a large-scale evaluation of the *MindBabyBody* program. Recruitment of a *universal* population has a range of benefits including maximum inclusivity of women who may later develop depression, with the potential to prevent the greatest number of future episodes of mental disorders in the general population. It has been argued that even small reductions in population prevalence of perinatal mental disorders have a greater public health benefit than treating individuals who are already symptomatic [50] and that a comprehensive approach to mental health must include both mental health promotion and attempts to prevent mental health problems [51]. However, recruitment of a *universal* population does not necessarily result in a representative sample. There is evidence in our study, and in others [22], that universal recruitment strategies result in samples with higher levels of depressive and anxiety symptoms than the general population. While baseline means for the *universal* population in our study were not above clinical cut-offs, over a third of women in the RCT scored above clinical cut-offs for depression, anxiety and stress - indicating a sample with more mental health symptoms than the general perinatal population [52].

A more targeted approach, utilising a *selected* population of women at risk of developing mental health problems also has a range of benefits, including that it may be more cost-effective, and will target resources where they are most urgently needed (i.e. women at greatest risk of mental health problems in pregnancy and the postnatal period). It has also been argued that universal interventions to improve overall public health have the potential to *increase* health inequalities [53]. Programs offered to all women are most likely to be taken up by the most highly-resourced and highly-motivated individuals, with vulnerable or at-risk women being less likely to participate. There was evidence of this is the current pilot, which recruited a relatively older and more educated population. In future trials, we will explore strategies that engage vulnerable populations, and may specifically target certain at-risk groups (such as young women, single women, and women of non-English speaking background).

### Recruitment

A mailed brochure at the time of booking-in yielded the highest number of participants in the RCT (n = 16), however 2500 brochures were mailed to achieve this number. A researcher based in the antenatal clinic (recruiting women face-to-face) yielded similar numbers over the study period (n = 14) but involved significant Research Assistant time, and a direct comparison of strategies is difficult. Childbirth education classes and physiotherapy classes were not successful recruitment strategies in this pilot, recruiting just two participants over the study period.

Anecdotal feedback from potential participants during the recruitment process has led us to consider amending the study brochure to use less clinical, more inclusive language. The use of words such as ‘*depression’* and ‘*anxiety’* may have been off-putting for some women, especially in a universal population. In addition, the provision of a group after hours (6-8 pm), as opposed to business hours, greatly increased the speed of recruitment, and will be considered essential in future trials. The sample recruited for the current pilot, though diverse in some ways, was likely not representative of the general population of antenatal patients who attend the Royal Women’s Hospital [54]. For example, our sample included a high number of women with a tertiary education, most were employed, and with a mean age of 33 years (range 19–45 years), and all were English-speaking. Future trials will explore strategies to engage a more diverse and representative sample.

### Retention

The biggest loss to follow-up in the study occurred due to delivery of babies prior to the completion of follow-up measures, and a key finding of the current pilot study is therefore the value of recruiting women early in pregnancy to reduce this loss. An important advantage of the brochure recruitment strategy was that it recruited women at their earliest contact with the hospital. In qualitative interviews, women provided further support for early recruitment, with several participants commenting that they would have preferred to complete the program earlier in pregnancy, to provide more opportunity to practice the mindfulness strategies before birth.

The retention rate in the current study was somewhat disappointing and future trials will include strategies aimed at increasing participant retention. As stated above, focusing recruitment strategies on the early periods of pregnancy will help to reduce some loss to follow-up. The two-armed nature of the current trial meant that questionnaire follow-up procedures were decentralised, and any future trial will utilise strengthened and centralised processes of questionnaire follow-up, to maximise questionnaire returns.

### Engagement with mindfulness practice

Data collected on formal and informal mindfulness practice (both qualitative and quantitative) indicates an adequate level of engagement with mindfulness practices. The most common level of formal practice was 3–4 days per week, and around half of participants practiced informal mindfulness on 4 days a week or more. Larger participant numbers will allow us to conduct stratified analyses of change in outcome data according to level of formal and informal practice during participation in the intervention, but with the current sample size this was not possible. Importantly, we also discovered that 50% of participants in the control arm of the RCT (‘care as usual’ group) had practiced prenatal yoga during their pregnancy. As a mindfulness-based practice, it is possible that this could cause some distortion of study findings, and this will be an important consideration for future RCTs.

### Acceptability and discriminant ability of study measures

The questionnaire in the current pilot study was quite repetitive, due to our desire to test different outcome measures, and we received anecdotal feedback from participants who found this annoying. There were no clear findings regarding the discriminant ability of measures, with all appearing to show change over time. Future decisions on outcome measures will be guided by the aim to reduce length and repetition in questionnaires, in order to improve participant retention.

### Outcome data

The outcome data presented must be interpreted with caution, as the study was designed to test feasibility and acceptability of outcome measures rather than changes in outcome. However, our findings show some indication of within group effects over time for women who took part in the *MindBabyBody* intervention. Significant improvements were noted for depression, anxiety and mindfulness scores for the selected population in the non-randomised study, and significant improvements were noted in anxiety and mindfulness scores in the randomised trial with a universal population. While the care as usual group in the randomised trial did not show any significant within group effects, there was no evidence of between group differences when comparing the intervention group to the care as usual group.

The qualitative component of the pilot (though based on a small and self-selected sample) supports the quantitative findings, indicating the potential of mindfulness practices to significantly improve women’s wellbeing during pregnancy. It provides evidence of the ways in which mindfulness can positively influence the lives of pregnant women (or some pregnant women). The interviews revealed that individuals engaged with the program in different ways, utilising varied levels of meditation, and were attracted to different types of mindfulness practice. Changes attributed to mindfulness practice included a reigning in of destructive patterns (cognitive, emotional or behavioural) which stopped challenging situations escalating. Flowing on from this, participants reported improvements to interpersonal relationships, sleeping patterns, and to mood and quality of life.

### Strengths and limitations

Some important limitations of the current pilot should be acknowledged when considering the findings. An accurate assessment of response rate was difficult to assess for all recruitment pathways. It was possible for women to be exposed to more than one recruitment method, and this potential cross-over of recruitment pathways is an acknowledged limitation of the pilot study, and may have resulted in some sample bias which would affect the clinical outcomes. However, as a pilot study, this limitation is unlikely to affect our intended aims of exploring feasibility issues. Only a small number of self-selected women took part in the qualitative interviews, and as such, we cannot generalise about the experiences of participants in the intervention more broadly. Because of the small number of participants in the pilot study, analyses regarding outcomes of the intervention should be interpreted with great caution. A larger sample size would have provided more robust findings, in regards to both feasibility and outcome data. We were unable to complete an intent-to-treat analysis on women who delivered their baby prior to follow-up, as their circumstances were too significantly changed to allow a comparison on outcome measures. A sample size estimate was not conducted on the pilot study data, given the advice of Kraemer and colleagues that this is not an accurate means of estimating the required sample size for a large randomised controlled trial [55].

Strengths of the study include: the comparison of three different recruitment methods to the RCT (including piloting of the randomisation process), comparison of a universal recruitment strategy to a targeted/at risk recruitment approach, a comparison of different standardised measures to assess the same outcome dimension, and the collection of both quantitative and qualitative data to assess feasibility and acceptability of recruitment, follow-up methods, and the intervention.

### Considerations for future trials

- Employ recruitment strategies to engage younger, less educated, and more ethnically diverse women.
- Implement strategies to increase participant retention - most notably undertaking recruitment as early as possible in pregnancy given that the most frequent reason for loss to follow-up was delivery of baby prior to follow-up.
- Explore face-to-face contacts which occur earlier in pregnancy (i.e. the first hospital visit).
- Reduce repetition in questionnaires, and aim to provide a more holistic picture of women’s health and wellbeing, drawing on learnings from large cohort studies which have achieved excellent retention rates over time [56].
- Consider the use of an active control group, to avoid participant disappointment in the randomisation process, and to account for group effects.
- Given the popularity of prenatal yoga (50% in the control group), careful measurement of this in future RCTs will be undertaken to assess the potential for bias.

## Conclusions

This small pilot study provides support for the feasibility of an antenatal mindfulness intervention to reduce psychological distress offered within the antenatal care services of a large tertiary maternity hospital. Recruitment through antenatal clinics, midwife referral, and a mailed brochure at the time of booking-in all yielded similar participant numbers over the study period. Recruitment through childbirth education classes and physiotherapy classes was not effective. We found preliminary evidence of the effectiveness of the intervention, with indications of improvement to self-reported depression and anxiety. Major challenges to address in future trials include: finding ways to facilitate recruitment in early pregnancy, and engaging younger women and other vulnerable populations.
